# Endovascular Treatment of May-Thurner Syndrome in an Office-Based Laboratory

**DOI:** 10.7759/cureus.63903

**Published:** 2024-07-05

**Authors:** Ryan Nolan, Harneet S Sangha, Edward J Arous

**Affiliations:** 1 School of Medicine, University of Nevada Reno, Reno, USA; 2 School of Medicine, Elson S. Floyd College of Medicine, Spokane, USA; 3 Department of Vascular Surgery, The Vascular Care Group, Worcester, USA

**Keywords:** venous stenting, endovascular intervention, outpatient procedure, office-based lab, deep vein thrombosis, compressive venous syndrome, may-thurner syndrome

## Abstract

May-Thurner syndrome (MTS) is a rare condition that increases the risk of left-sided iliofemoral venous thrombosis due to compression of the left common iliac vein by the right common iliac artery. Treatment for symptomatic MTS typically includes combined anticoagulation and endovascular therapy. This patient presented to the emergency department with acute left lower extremity pain and swelling. After imaging confirmed MTS, the patient was discharged from the ED and expeditiously treated in an office-based lab (OBL) setting with venous thrombectomy, angioplasty, and stenting. The setting where endovascular therapy is performed may significantly impact access to care for patients. Additionally, cost-effectiveness is a factor that should be considered when deciding the treatment site of service. We demonstrate the safety and cost-viability of performing venous thrombectomy, angioplasty, and stenting in an outpatient setting for the treatment of acute iliofemoral venous thrombosis.

## Introduction

May-Thurner syndrome (MTS) is a rare condition that increases the risk of left-sided iliofemoral venous thrombosis due to the compression of the left common iliac vein by the right common iliac artery [1]. Generally, the condition is clinically asymptomatic due to the presence of collateral venous drainage or mild compression [1,2]. It is predicted that the prevalence of this condition is underreported, indicating it may be an underappreciated risk factor for iliofemoral venous thrombosis and is expected to cause between 2% and 5% of deep vein thrombosis (DVT) [3]. The compression and pulsatile irritation of the left common iliac vein result in the formation of fibrous banding in the lumen wall (sometimes referred to as spurs).

Patients remain asymptomatic due to the formation of venous collaterals, preventing a critical obstruction. MTS may become symptomatic when precipitated by events such as surgery or pregnancy, although this is not always the case [1]. Initial symptoms are generally due to the formation of a DVT and include painful left lower extremity swelling, localized warmth, cramping, leg heaviness, venous ulcers, and varicose veins [1,2]. However, there are some patients who may present on a wider spectrum of presentations that represent chronic venous insufficiency [2]. MTS should be suspected in patients with unprovoked acute or recurrent proximal DVT, especially if they have risk factors such as female sex, oral contraceptive use, and young age [1,2].

Diagnosis of MTS may be made during a work-up of DVT or following suspicion of MTS due to clinical presentation and risk factors. If the provider is suspicious of DVT, it is necessary to evaluate with color Doppler ultrasound, but this is insufficient for the diagnosis of MTS, which requires cross-sectional ilio-caval imaging [4,5]. Standard imaging for MTS includes magnetic resonance (MR) venography and intravascular ultrasound (IVUS) [5]. Previously, the standard imaging had been MR venography with multi-detector computed tomography (MDCT) venography [5,6].

The current standard of care for symptomatic MTS involves a combination of immediate anticoagulation and subsequent endovascular intervention. Endovascular care is provided with venography, IVUS-guided angioplasty, and stenting of the left common iliac vein [4]. Pulmonary embolism (PE) is a concern whenever a DVT is present; however, due to the venous compression that is characteristic of MTS, research has shown that the incidence of PEs in these patients is significantly lower compared to the typical PE risk with a DVT [2]. Therefore, placement of an inferior vena cava filter is not recommended for these patients. While intervention involving angioplasty and stenting is the mainstay for most MTS patients, another lesser-used procedure is catheter-directed thrombolysis, which is typically followed by stenting [4,5]. In asymptomatic patients, blood thinners and supportive measures such as compression stockings can be used [4,5].

## Case presentation

A 30-year-old female who is an active cigarette smoker presented to the emergency department with acute left lower extremity pain and swelling. A duplex ultrasound demonstrated acute occlusive deep venous thrombosis of the left common iliac through the left popliteal veins. A CT scan demonstrated a severe stenosis of the left common iliac vein. Five days after symptom onset, she was taken to the office-based lab (OBL), and access was performed via the left femoral vein.

**Figure 1:**
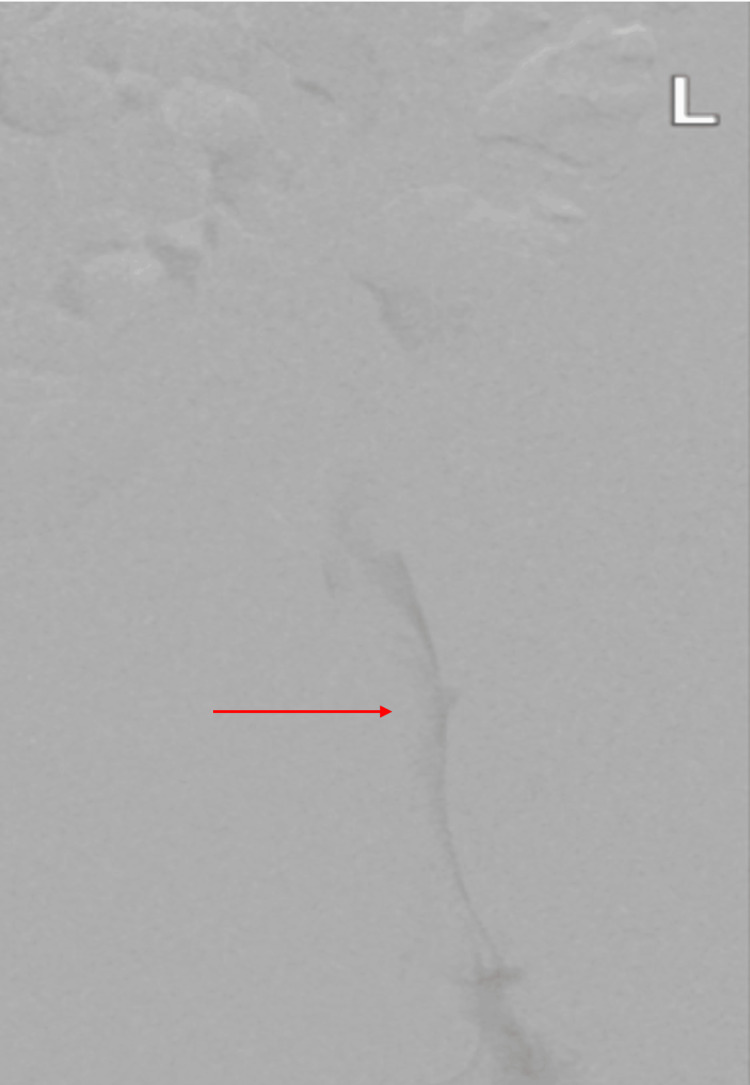
Initial venogram demonstrating acute left iliofemoral deep venous thrombosis (arrow), evidenced by decreased intravenous contrast.

**Figure 2:**
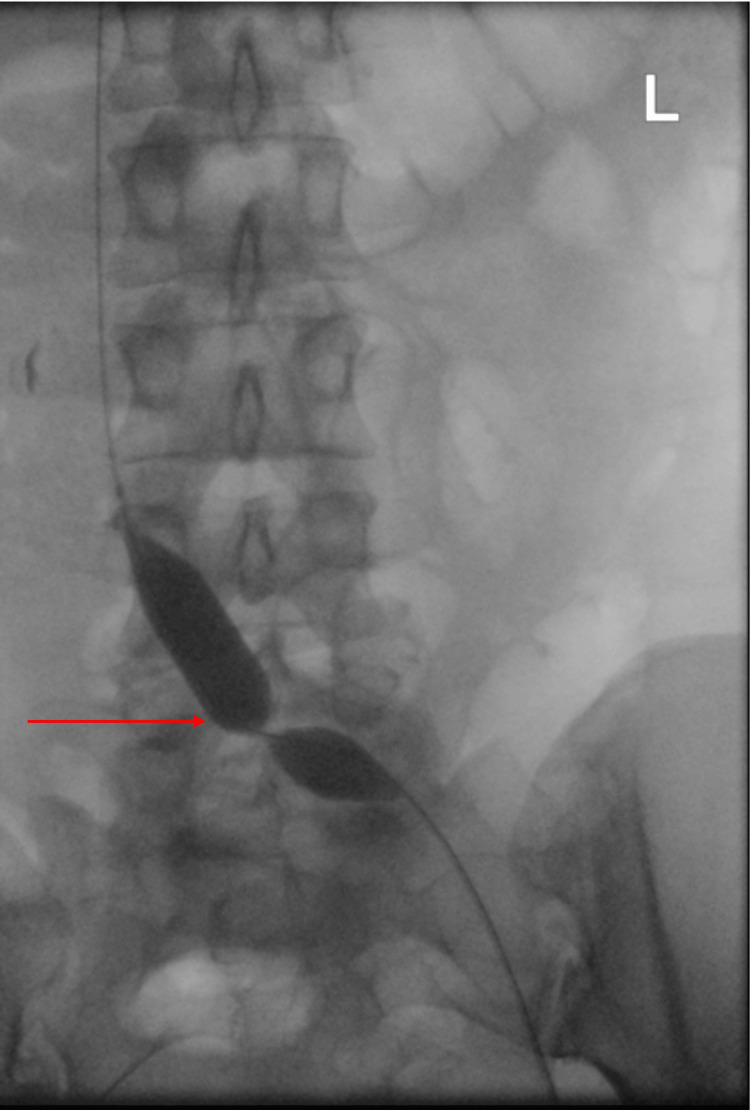
Balloon angioplasty at the site of the compression.

**Figure 3:**
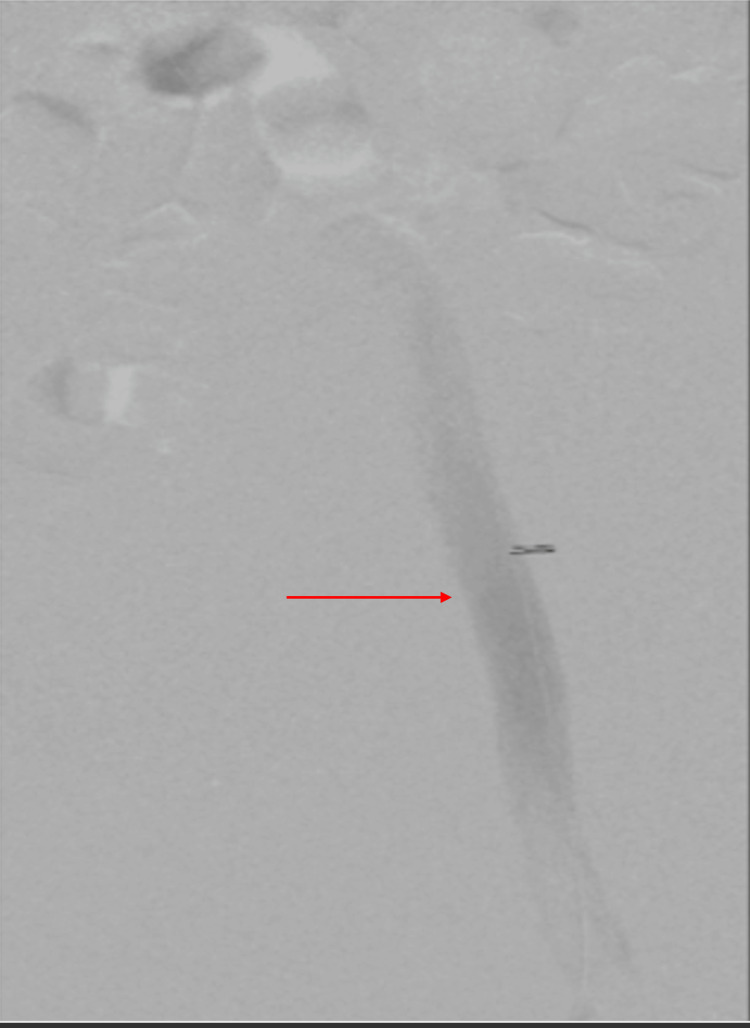
Venogram of the left iliofemoral segment following thrombectomy and balloon angioplasty, demonstrating recanalization (arrow).

The initial venography and intravascular ultrasonography demonstrated occlusion and acute thrombosis (Figure 1). Subsequently, venous thrombectomy was performed using a Philips Quickclear 10-french thrombectomy device. Balloon angioplasty demonstrated significant compression of the left common iliac vein at the site of arterial compression (Figure 2). Following thrombectomy and balloon angioplasty, Medtronic Abre stents were placed from the distal IVC to the left common femoral vein (Figure 3). The patient was discharged home on therapeutic apixaban after the procedure. The following day, the patient reported complete resolution of her left leg edema with no residual symptoms and no reported adverse reaction to the procedure.

## Discussion

MTS is likely to be an underdiagnosed condition that increases the risk of DVT, typically on the left side [1]. Systemic anticoagulation alone is not the standard of care for these patients, as it has been shown that anticoagulation alone may lead to recurrent DVT [4,5]. Therefore, endovascular therapy is essential for safely managing these patients. Because endovascular treatment is a mainstay in the treatment of MTS, these patients are likely to be suitable candidates for treatment in an OBL or other outpatient setting.

The setting in which a procedure is performed on a patient has a significant impact on post-operative recovery as well as costs to the patient and healthcare system [7]. When possible, it is likely safer for the patient if procedures, especially those such as endovascular procedures, can be performed on an outpatient basis [7]. In a national registry exploring the safety and quality of care for patients treated in OBLs, it was shown that procedures in this setting had a low complication rate of 1.87%, a major adverse event rate as low as 0.51%, and a hospital transfer rate of 0.62% [8]. OBLs are suited for patients who are receiving procedures that are considered low or moderate procedural risk. This includes a wide variety of endovascular procedures for both venous and arterial cases.

This case demonstrates that a patient with an acute occlusive DVT can be safely and comprehensively treated in an OBL. In the case of MTS, endovascular treatment is highly preferred over surgical thrombectomy. In fact, surgical thrombectomy treatment has only accounted for roughly 4% of MTS cases since 2000 [5]. In order to increase awareness of what OBLs can safely and cost-effectively offer, it is important to identify procedures that can be performed in OBLs and ensure that patients are made aware. It provides those who are hesitant to undergo a minimally invasive procedure due to hospital stays and associated costs with a procedural option.

Considerations that may support an inpatient procedure over the setting of an OBL or other outpatient center include significant recovery demands [8]. However, in endovascular and interventional care that have low recovery times and low complication rates, outpatient settings are likely more convenient and safe for patients. The implementation of OBLs also allows better utilization of hospital rooms for more complicated procedures and patients [7,8]. For surgical centers that see large volumes and have overscheduled ORs, it is beneficial to have OBLs nearby that can perform procedures that are low or moderate procedural risk.

## Conclusions

Our case report demonstrates the safety and efficacy of office-based angioplasty and stenting for acute occlusive DVT secondary to MTS. After the patient presented to the emergency department and was appropriately managed, her occlusion was successfully treated with endovascular standard of care in an OBL. The increase in the prevalence of ambulatory care settings for mild to moderately complex procedures highlights a trend in the shift of these procedures away from inpatient hospital settings. While the decision to perform procedures in an outpatient setting will vary based on patient complexity, provider comfort, and the condition itself, there is a clear potential benefit to expanding the settings in which patients may have a procedure performed. These benefits include increased scheduling flexibility, reduced healthcare costs, decreased hospital burden, and a decreased risk of in-hospital infections.
